# FlexBurst360 therapy – A new spine cord stimulation system (SCS) for patients with chronic pain

**DOI:** 10.1016/j.amsu.2022.104627

**Published:** 2022-09-13

**Authors:** Yumna Khabir, Adam Ali Asghar, Mahnoor Rehan Hashmi

**Affiliations:** Department of Medicine, Dow University of Health Sciences, Mission Rd, New Labour Colony Nanakwara, Karachi, Sindh, Pakistan

On Aug 23rd, 2022 Food and Drug Administration (FDA) approved the FlexBurst360 therapy incorporated into Abbott Laboratories' new spine cord stimulation system (SCS) for patients with chronic pain [1]. The FlexBurst360 therapy is the next generation of Abbott's proprietary BurstDR stimulation [1]. This product is designed to provide pain relief in up to six areas of the trunk and/or limbs. Programming adjusts according to a person's therapeutic needs. Proclaim Plus SCS is a battery-free system with a battery that can last up to 10 years [1]. It was noted that spinal cord stimulation provided immense relief for chronic pain patients [1].

Chronic pain affects more than 30% of people worldwide, placing a significant personal and financial strain on them [2]. As described by the International Association for the Study of Pain (IASP), chronic pain is a sensory and emotional experience that is associated with or similar to actual or potential tissue damage [2]. Most people seek medical attention because of pain, and three of the top 10 causes are osteoarthritis, back pain, and headaches. Among the four leading causes of years lost to disability, three of these (back pain, musculoskeletal disorders, and neck pain) are chronic pain conditions [2].

Chronic pain also has significant financial expenses. According to a 2010 Institute of Medicine report, chronic pain affects one in three Americans and costs the country between $560 and $635 billion annually in medical expenses and lost productivity. The price of caring for those who are institutionalized (such as inmates or nursing home patients), military personnel, children, or the expenses related to caregiving were not included in this estimate [2]. Recent studies show the average cost of chronic pain for one of the 15% of Australians living with it ranges from AU$22588 to $42979 when non-financial factors are taken into account [2].

The incidence of chronic pain in the undomiciled ranges from 47% to 63% according to research examining its impact on people's capacity to work and can have financial repercussions, including homelessness [2]. Chronic pain is one of the primary causes of disability. In addition to affecting relationships and self-esteem, chronic pain also increases the chances of divorce and suicide, as well as substance abuse [2]. Chronic pain is associated with a shorter life expectancy after adjusting for other factors [2].

The different neurostimulation techniques modulate pain pathways of body. Pain in the body is processed through ascending lateral and medial (pain evoking) and descending (pain inhibiting) pathways. The ascending pathways use C, Aδ, and Aβ-fibers to conduct pain information from ventral posterolateral nuclei of thalamus to the somatosensory cortex. Descending pain inhibitory pathway utilizes rostral ad pregenual anterior cingulate cortex.

The already in use, Tonic SCS act on concept of Gate Control Theory by Melzack and Wall [3]. The electrical impulses generated by SCS stimulate Aβ fibers in the dorsal column which activate inhibitory interneurons in the spinal dorsal horn [3]. These interneurons alter incoming nociceptive input from Aδ and C fibers and release the inhibitory neurotransmitter γ-aminobutyric acid (GABA), thereby ‘closing the gate’ and inhibiting pain sensations [3]. It is given at a frequency of 40–80 Hz and a pulse width ranging between 200 and 500 μs and at intensities which produce tingling sensations or paresthesias [3]. Tonic SCS has been clinically successful in improving chronic neuropathic pain syndromes but present with concerning limitations like insufficient pain relief and uncomfortable paresthesias [3]. These shortcomings pave way for the development of new strategies like Burst SCS.

Burst SCS is a neurostimulation device which delivers high frequency charge pulses at low altitudes [4]. These charges have pulse-free interphase delays between them and eventually a passive recharge (discharge) phase which allows recovery [4]. Burst SCS mimics the naturally occurring burst firing patterns of pain present in the body. Compared to the previous tonic SCS that only affects lateral and descending pathways, Burst SCS affects lateral, medial and descending pathways which explains more positive results shown by Burst SCS [4]. The action mechanism of Burst SCS shows that it activates GABAergic interneurons in the dorsal spinal horn and supraspinal areas [3]. Supraspinal areas are involved in motivation and emotions which are also activated by the use of Burst SCS. In this aspect Burst is superior to other forms of SCS due its effect not only on sensory and discriminative aspects of pain but also on emotional and motivational aspects [3]. A randomized crossover study demonstrated the use of burst SCS in suppressing chronic pain. Patients were assessed with fluorodeoxyglucose positron emission tomography (FGD-PET) and pain vigilance and awareness questionnaire. The result of the study showed that burst SCS modulates dorsal anterior cingulate gyrus more than tonic stimulation which altered patients pain perception [4].

Taking into account the burden chronic pain holds, it is important to conduct more extensive studies on the newly established FlexBurst360 therapy in order to know more about its indications and recommendations.

## Sources of funding

The authors have no financial or proprietary interest in the subject matter of this article.

## Ethical approval

The approval was not needed.

## Consent

No consent was required.

## Author contribution

Yumna Khabir; Conceptualization, literature review and manuscript writing.

Adam Ali Asghar; Literature review and manuscript writing.

Mahnoor Rehan Hashmi; Literature review and manuscript writing.

## Registration of research studies

1. Name of the registry:
2. Unique Identifying number or registration ID:
3. Hyperlink to your specific registration (must be publicly accessible and will be checked):

## Guarantor

Yumna Khabir, Adam Ali Asghar and Mahnoor Rehan Hashmi.

## Declaration of competing interest

The authors have no conflict of interest.
